# Serum S100 Protein Is a Reliable Predictor of Brain Injury After Out-of-Hospital Cardiac Arrest: A Cohort Study

**DOI:** 10.3389/fcvm.2021.624825

**Published:** 2021-02-09

**Authors:** Martin Kleissner, Marek Sramko, Jan Kohoutek, Josef Kautzner, Jiri Kettner

**Affiliations:** ^1^Department of Cardiology, Institute for Clinical and Experimental Medicine (IKEM), Prague, Czechia; ^2^Third Faculty of Medicine, Charles University, Prague, Czechia; ^3^First Faculty of Medicine, Charles University, Prague, Czechia; ^4^Palacky University Medical School, Olomouc, Czechia

**Keywords:** brain injury, cardiac arrest, hypothermia, neuroprognostication, prehospital resuscitation, acute cardiac care

## Abstract

**Purpose:** To evaluate serum S100 protein at hospital admission and after 48 h in early neuroprognostication of comatose survivors of out-of-hospital cardiac arrest (OHCA).

**Methods:** The study included 48 consecutive patients after OHCA, who survived for at least 72 h after the event. The patients were divided based on their best cerebral performance category (CPC) achieved over a 30 day follow-up period: favorable neurological outcome (CPC 1–2) vs. unfavorable neurological outcome (CPC 3–4). Predictors of an unfavorable neurological outcome were identified by multivariable regression analysis. Analysis of the receiver operating characteristic curve (ROC) was used to determine the cut-off value for S100, having a 0% false-positive prediction rate.

**Results:** Of the 48 patients, 30 (63%) had a favorable and 18 (38%) had an unfavorable neurological outcome. Eleven patients (23%) died over the 30 day follow-up. Increased S100 levels at 48 h after OHCA, but not the baseline S100 levels, were independently associated with unfavorable neurological outcome, with an area under the ROC curve of 0.85 (confidence interval 0.74–0.96). A 48 h S100 value ≥0.37 μg/L had a specificity of 100% and sensitivity of 39% in predicting an unfavorable 30 day neurological outcome.

**Conclusion:** This study showed that S100 values assessed 48 h after an OHCA could independently predict an unfavorable neurological outcome at 30 days.

## Introduction

Out-of-hospital cardiac arrest (OHCA) is defined as the cessation of cardiac mechanical activity as confirmed by the absence of signs of circulation occurring outside the hospital (1). Coronary artery disease with initial ventricular fibrillation and subsequent degeneration into asystole is a leading cause of non-traumatic OHCA (2, 3). OHCA remains a major health problem, as it frequently results in patient death or severe neurological disability, even with early resuscitation efforts and high-quality intensive care (4–7). Prolonged care for unconscious patients with severe brain injury and little hope of a meaningful life is costly and burdensome both for relatives and medical staff. Early discontinuation of intensive treatment should, therefore, be considered in selected cases with a grave neurological prognosis. However, an early determination of the patient's prognosis is difficult and often biased by unavoidable patient sedation (8).

To improve early predictions of neurological outcomes after an OHCA, studies have used various serum biomarkers of neuronal injury (9). Advantages of these biomarkers over other prognostic modalities and clinical examination include quantitative results and independence from the effects of sedatives. Their disadvantage is the lack of a consistent threshold for identifying patients destined to a poor outcome with a high degree of certainty (10). Astroglial S100 calcium-binding protein has been shown to correlate with the extent of nerve tissue damage and neurological outcomes (10). The prognostic value of S100 has been investigated in several studies; however, most of them suffered from significant limitations: (1) they included mixed populations of OHCA and in-hospital cardiac arrest patients (11–16); (2) targeted temperature management (TTM), which is the currently recommended therapy with demonstrated neuroprotective effects, was not routinely used (16–26); (3) they included all-cause mortality as a part of a combined clinical endpoint (12, 26, 27), or the causes of death were not analyzed (13–15, 21–25, 28–30), which imposed a significant bias since S100 levels reflect brain injury/death but not death from other causes.

To avoid the above-mentioned limitations, we conducted this prospective study to verify the validity of baseline and 48 h serum S100 levels as a predictor of the best neurological outcome during the 30 day period after a non-traumatic OHCA.

## Materials and Methods

### Study Design and Population

**Figure 1:**
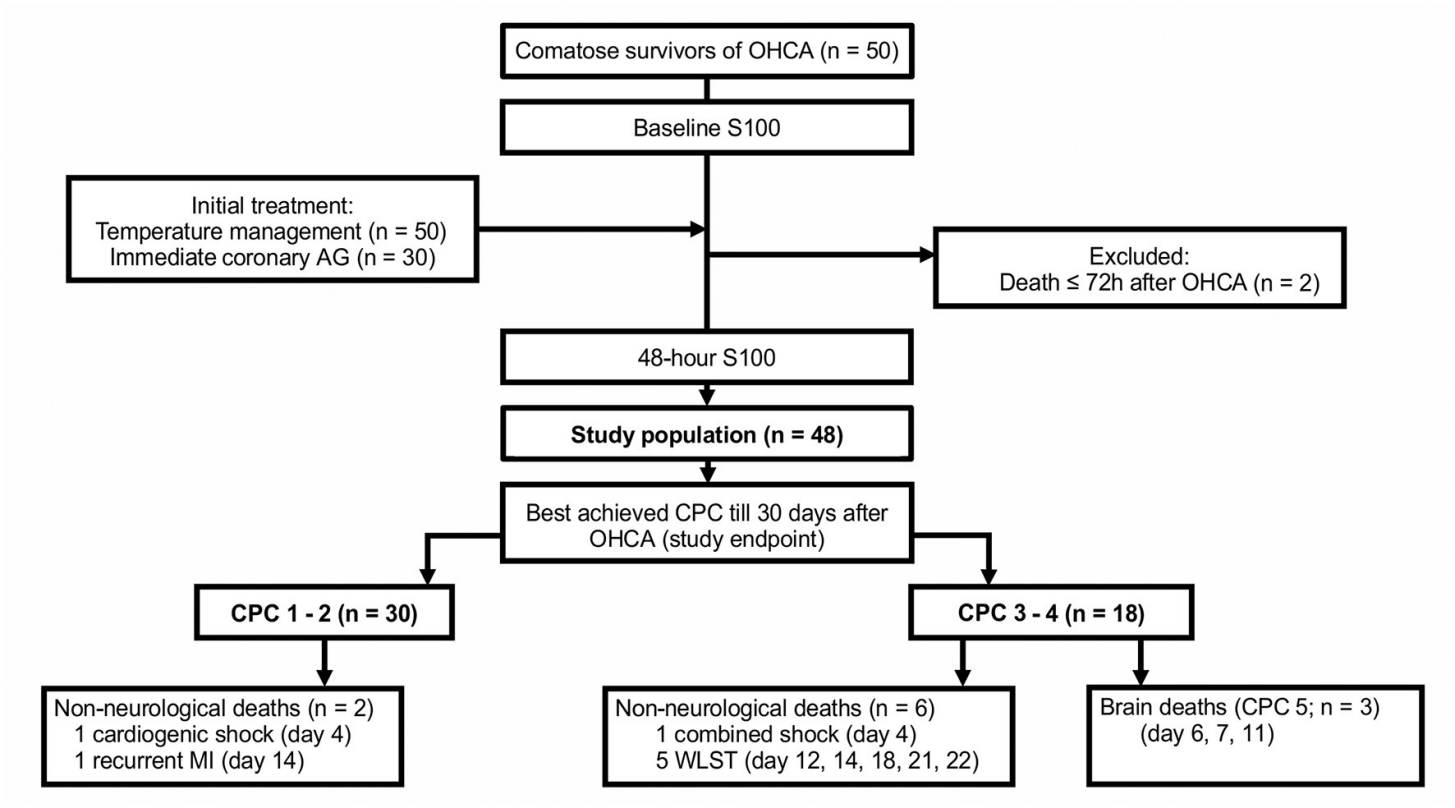
The study flowchart. AG, angiography; CPC, cerebral performance category; MI, myocardial infarction; OHCA, out-of-hospital cardiac arrest; WLST, withdrawal of life-sustaining treatment.

This was a prospective, single-center study conducted in a high-volume cardiology intensive care unit (CICU) of a tertiary center. The analyzed sample included 48 of 50 consecutive patients after an OHCA who underwent TTM at our CICU and survived at least 72 h after admission (Figure 1). The study was approved by the institution's ethics committee (approval #G-18-62); patient informed consent was waived since blood samples were obtained during clinically indicated routine blood collections. Clinical data were recorded prospectively using a predefined structured form. The choice of the recorded variables followed recommendations for uniform reporting of OHCA (i.e., the Utstein Style) (31).

In our region, all ambulance crews arriving at the scene were staffed by a physician. After recovery of spontaneous circulation, the ambulance physician consulted, by phone, with the emergency physician on duty at our CICU. Patients with a suspected cardiac cause, or in cases where the cause of the OHCA was not immediately evident, were transported to our CICU with the prospect of coronary angiography and other specific cardiological exams, whereas patients with an evident non-cardiac cause were usually referred to general intensive care units at other institutions (32).

According to our protocol, TTM was initiated in all patients immediately after admission. Cooling was achieved using a hypothermia water blanket (PlastiPad, Blanketrol system, Cincinnati Sub-Zero, USA), ice packs, and cold infusions. Body temperature was measured using a thermistor-tipped urinary catheter; the target was set for between 32 and 36°C for the first 12–24 h (33–35). Patients with evident or probable acute coronary syndrome then underwent immediate coronary angiography (<2 h after admission), which was available 24/7. In the remaining patients, coronary angiography was performed after completion of TTM and after verification of a favorable neurological status. Patients received standard cardiology and intensive care management according to the guidelines (10).

### Protein S100 Analysis

Serum for the determination of S100 levels (S100 A1B and S100 BB) was obtained immediately after hospital admission (baseline) and again at 48 h. The samples were stored at −70°C until batch analysis, which was done using a commercially available electrochemiluminescence immunoassay (Elecsys S100®, Roche Diagnostics, Mannheim, Germany). The detection range of the test was 0.005–39 μg/L with an inter- and intra-assay coefficient of variation of 5.6 and 2.3%. The laboratory staff performing the biomarker analysis was blinded to the clinical results.

### Outcome

The primary study endpoint was the best achieved cerebral performance category (CPC) between hospitalization day 3 and day 30. CPC 1 (good cerebral performance) and CPC 2 (moderate cerebral disability) were regarded as favorable neurological outcomes, while CPC 3 (severe cerebral disability) and CPC 4 (coma or vegetative state) were considered as unfavorable neurological outcomes. In patients who died, the exact cause of death was determined (Figure 1); only brain death being CPC 5 (36). Clinical follow-up CPC data after discharge from the index hospitalization were obtained by outpatient visits (*n* = 24), by phone calls with the patient, the patient's relative, or a general practitioner (*n* = 18), or from discharge letters from other hospitals (*n* = 3). End-of-life decisions were not made at our institution; patients with persistent severe neurological deficits were transferred to chronic care units.

### Statistical Methods

Pre-study sample size calculations were performed using the OpenEpi online calculator (37) and were based on day 2 serum levels of S100 published by Prohl et al. (11). A sample size of 50 subjects was estimated to achieve a power of 90% for a two-sided test at a level of α = 0.05.

Comparisons between patients, relative to best achieved favorable (CPC 1–2) vs. unfavorable neurological (CPC 3–4) outcomes were performed using the Student's *t*-test, Wilcoxon/Kruskal-Wallis test, Chi-Square test, or Fisher's exact test, as appropriate. Odds ratios and confidence intervals (CI) were computed, and the Haldane-Anscombe correction was used in the case of a zero cell in the contingency tables. Factors associated with dichotomized neurological outcomes were identified using univariate, and subsequently, multivariate logistic regression analysis, which included statistically relevant (*p* < 0.1) univariate predictors. The predictive value of S100 was determined using the receiver operating characteristic. The 95% binomial CIs for false-positive rates were defined using the Blyth-Still-Casella method. The analyses were performed using JMP 10 statistical software (SAS Institute Inc., Cary, USA, RRID:SCR_014242) and R version 4.0.0 (www.r-project.org, Vienna, Austria, RRID:SCR_001905). A *p* < 0.05 was considered significant.

## Results

Of the 48 patients, a total of 30 (63%) had a favorable neurological outcome (CPC 1–2), while 18 (38%) had an unfavorable neurological outcome (CPC 3–4) 30 days after the OHCA. Eleven patients (23%) died, of those, eight died from irreversible brain damage. A comparison of patient clinical characteristics, based on the best achieved 30 day neurological outcome, is shown in Table 1.

**Table 1:** Clinical characteristics of the study population overall and relative to neurological outcomes.

	**All patients**	**Best CPC within 30 days**		**Univariate analysis**		**Multivariate analysis**	
		**CPC 3–4**	**CPC 1–2**	**Odds ratio**	***p*-Value**	**Odds ratio**	***p*-Value**
	***n* = 48**	***n* = 18**	***n* = 30**	(95% CI)^*^		(95% CI)^*^	
**Clinical characteristics**							
Age (years)	61 ± 12	64 ± 11	59 ± 13	1.0 (1.0–1.1)	0.22		
Male	40 (83)	17 (94)	23 (77)	5.2 (0.6–46)	0.23		
**Comorbidities**							
Diabetes mellitus	7 (15)	2 (11)	5 (17)	0.6 (0.1–3.6)	0.70		
Arterial hypertension	31 (65)	13 (72)	18 (60)	1.7 (0.5–6.1)	0.39		
Smoking	22 (46)	7 (39)	15 (50)	0.6 (0.2–2.1)	0.45		
Dyslipidemia	16 (33)	7 (39)	9 (30)	1.5 (0.4–5.1)	0.53		
Coronary artery disease	12 (25)	6 (33)	6 (20)	2.0 (0.5–7.5)	0.30		
**Biomarkers**							
Lactate at baseline (mmol/L)	3.2 [2.6–5.7]	4.3 [2.8–6.6]	2.9 [2.2–5.8]	1.2 (1.0–1.5)	0.11		
Hs troponin T at baseline (ng/L)	126 [49–260]	157 [44–352]	123 [49–226]	1.0 (1.0–1.0)	0.38		
S100 at baseline (μg/L)	2.0 [1.0–3.8]	2.6 [1.0–4.2]	1.9 [1.0–3.3]	1.0 (1.0–1.1)	0.28		
S100 48 h (μg/L)	0.1 [0.1–0.3]	0.3 [0.1–1.0]	0.1 [0.1–0.1]	2.7 (1.5–6.2)	<0.0001	1.6 (1.1–4.0)	<0.01
S100 48 h ≥ 0.37 μg/L	7 (15)	7 (39)	0 (0)	40 (2.1–754)	<0.001		
S100 decrease (μg/L)	1.9 [0.8–3.5]	2.1 [0.5–3.7]	1.9 [1.0–3.2]	1.0 (1.0–1.0)	0.94		
**Left ventricular ejection fraction (%)**	36 ± 13	41 ± 13	33 ± 11	1.1 (1.0–1.1)	0.02	1.1 (1.0–1.2)	0.07
**Resuscitation-related characteristics**							
Witnessed OHCA	43 (90)	18 (100)	25 (83)	8.0 (0.4–153)	0.14		
Basic life support performed	40 (83)	15 (83)	25 (83)	1.0 (0.2–4.8)	1.00		
Basic life support time (min)	9 [7–11]	10 [5–12]	9 [8–10]	1.0 (0.8–1.3)	0.51		
VT/VF upon first contact	38 (79)	10 (56)	28 (93)	0.1 (0.0–0.5)	<0.01	0.1 (0.0–1.1)	0.06
Advanced life support time (min)	17 [10–21]	17 [15–24]	17 [8–22]	1.0 (1.0–1.1)	0.38		
Time from OHCA to ROSC (min)	22 [17–30]	24 [20–32]	21 [15–30]	1.0 (1.0–1.1)	0.22		
Normal pupillary reflex at baseline	45 (94)	17 (94)	28 (93)	1.2 (0.1–14)	1.00		
**Electrocardiography**							
ST-segment elevation or LBBB	24 (50)	10 (56)	14 (47)	1.4 (0.4–4.6)	0.55		
ST-segment depression	18 (38)	10 (56)	8 (27)	3.4 (1.0–12)	0.05	3.8 (0.6–29)	0.15
Right bundle branch block	6 (13)	4 (22)	2 (7)	4.0 (0.7–25)	0.18		
No ischemic ECG changes	9 (19)	3 (17)	6 (20)	0.8 (0.2–3.7)	1.00		
**Temperature management**							
Body temperature at baseline (°C)	35.7 ± 0.8	35.5 ± 0.8	35.8 ± 0.7	0.7 (0.3–1.5)	0.34		
Body temperature during TTM (°C)	34.4 ± 0.6	34.4 ± 0.6	34.4 ± 0.6	1.0 (0.4–3.0)	0.95		
Time from OHCA to target range (min)	99 [68–172]	96 [74–158]	114 [51–199]	1.0 (1.0–1.0)	0.88		
Time from ROSC to target range (min)	73 [40–154]	60 [39–120]	88 [45–167]	1.0 (1.0–1.0)	0.30		
Time within the temperature range (h)	22 [15–26]	25 [17–27]	22 [14–25]	1.1 (1.0–1.2)	0.13		
**Cardiology intensive care unit care**							
Revascularization immediate (≤ 2 h)	18 (38)	8 (44)	10 (33)	1.6 (0.5–5.3)	0.44		
Coronary angiography not performed	6 (13)	4 (22)	2 (7)	4.0 (0.7–25)	0.18		
Need for vasopressors	25 (52)	13 (72)	12 (40)	3.9 (1.1–14)	0.03	3.2 (0.4–26)	0.25
Length of stay (days)	6 [4–8]	5 [3–6]	7 [5–9]	0.8 (0.6–1.0)	0.13		
**Final diagnosis**							
Myocardial infarction	32 (68)	11 (65)	21 (70)	0.8 (0.2–2.8)	0.71		
Cardiomyopathy	4 (8)	0 (0)	4 (13)	0.2 (0.0–3.1)	0.28		
Primary arrhythmogenic cause	2 (4)	0 (0)	2 (7)	0.3 (0.0–6.8)	0.52		
Extra-cardiac	5 (10)	4 (22)	1 (3)	8.3 (0.8–81)	0.06	0.7 (0.0–19)	0.80

The values are count (%), median [Q1–Q3], or mean ± standard deviation. Factors associated with neurological outcomes at a significance level of p < 0.1 in the univariate analysis were also tested in the multivariate analysis.

* *Odds ratios and confidence intervals were calculated for unfavorable neurological outcomes. For continuous variables, unit odds ratios for 1.0 increments with corresponding confidence intervals are given. However, for S100, unit odds ratios and confidence intervals were calculated for 0.1 increments. CI, confidence interval; CPC, cerebral performance category; ECG, electrocardiogram; hs, high sensitivity; LBBB, left bundle branch block; OHCA, out-of-hospital cardiac arrest; ROSC, return of spontaneous circulation; TTM, targeted temperature management; VT/VF, ventricular tachycardia/fibrillation*.

**Figure 2:**
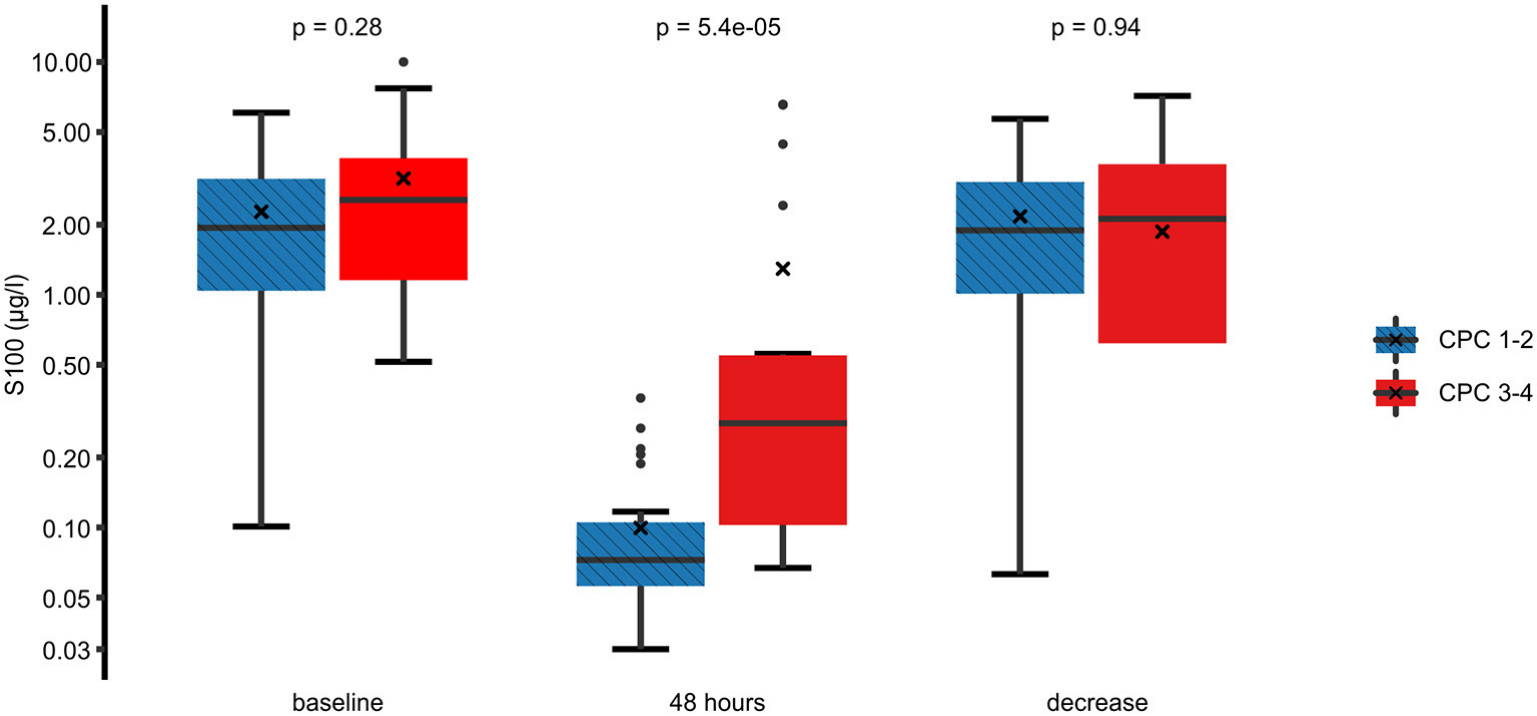
Comparison of the S100 levels between a favorable and an unfavorable neurological outcome. Best (numerically lowest) CPC score between day 3 and day 30 was determined and dichotomized into CPC 1–2 vs. 3–4. Box plots for S100 at baseline, 48 h, and the difference between these time points are shown on a logarithmic scale. Crosses (×) indicate the mean value. CPC, cerebral performance category.

In the univariate analysis, an unfavorable neurological outcome was associated with higher 48 h S100 levels, higher initial left ventricular ejection fraction, absence of a shockable rhythm at first medical contact, the presence of ST-segment depressions on the first electrocardiogram, and the need for vasopressors during the 24 h following the OHCA. On the other hand, there was no significant association between neurological outcomes and baseline S100 levels or between the absolute decrease in S100 between baseline and 48 h (Figure 2). Furthermore, there was no relationship between neurological outcomes and the time delay from OHCA to the start of TTM or to the achievement of the target temperature.

**Figure 3:**
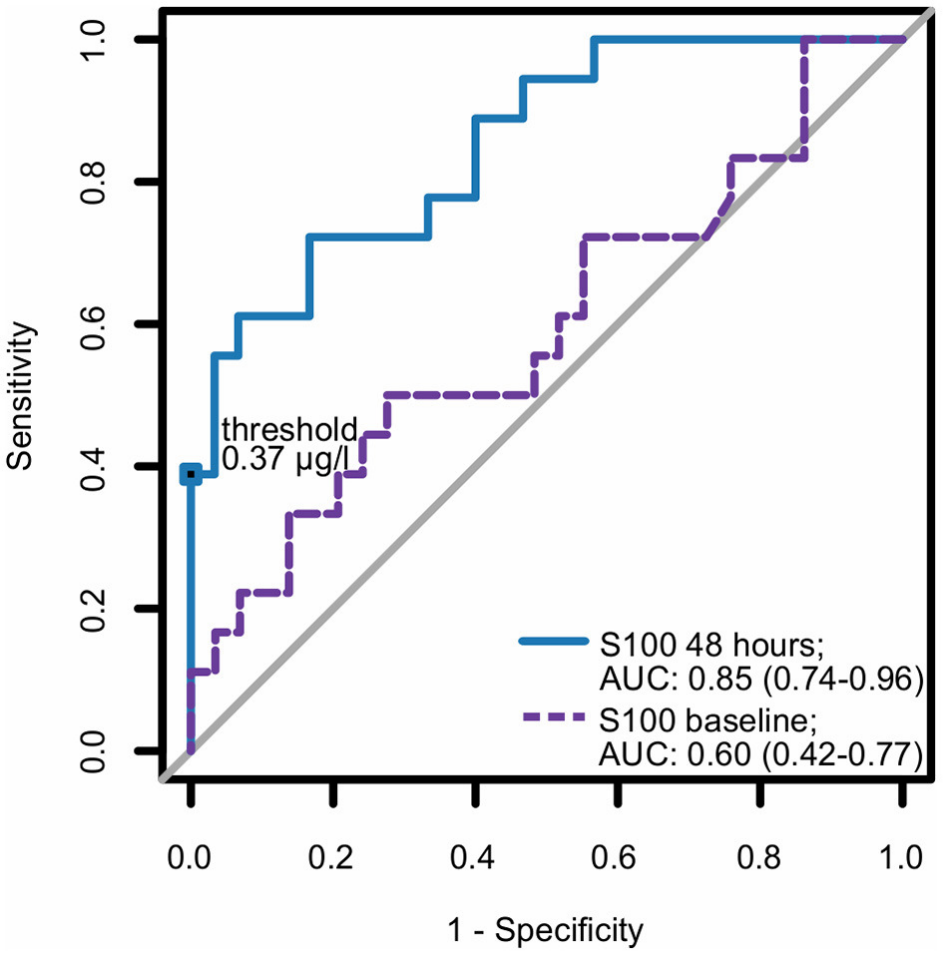
ROC curve for S100 as a predictor for neurological outcome. Best (numerically lowest) CPC score between day 3 and day 30 was determined and dichotomized into CPC 1–2 vs. 3–4. Consequently, the ROC curve of S100 at baseline and at 48 h for the dichotomized neurological outcome was plotted and corresponding area under the curve (AUC) with 95% confidence interval (in parentheses) was calculated. CPC, cerebral performance category; ROC, receiver operating characteristic.

In the multivariate analysis, increased S100 levels at 48 h were the only independent predictors of an unfavorable neurological outcome up to 30 days. The area under the receiver operating characteristic curve for S100 levels at 48 h, used for prediction of neurological outcomes, was 0.85 (CI 0.74–0.96) (Figure 3). At 48 h, S100 levels above the cut-off value of ≥0.37 μg/L had 100% specificity and 39% sensitivity for predicting an unfavorable 30 day neurological outcome (Table 2), while a cut-off value of 0.12 μg/L, determined as the Youden index, provided an unacceptably high false-positive rate for unfavorable outcomes (17%).

**Table 2:** Cut-off values of 48 h serum S100 levels for unfavorable neurological outcome.

**Cut-off (μg/l)**	**FPR %** **[95% CI]**	**Specificity %** **[95 % CI]**	**Sensitivity % [95% CI]**
≥0.12	17 [6–34]^*^	83 [66–94]	72 [47–89]
≥0.21	13 [4–30]	87 [70–96]	61 [37–82]
≥0.22	10 [2–26]	90 [74–98]	61 [37–82]
≥0.27	7 [1–21]	93 [79–99]	56 [33–77]
≥0.36	3 [0–17]	97 [83–100]	39 [18–63]
≥0.37	0 [0–11]	100 [89–100]	39 [18–63]

Best (numerically lowest) CPC score between day 3 and day 30 was determined and dichotomized into CPC 1–2 vs. 3–4. Consequently, the discrimination ability of S100 at 48 h for CPC 3–4 was analyzed.

* *Youden index. CI, confidence interval; FPR, false positive rate*.

Table 3 provides univariate analysis of clinical characteristics relative to 48 h S100 cut-off value of 0.37 μg/L. Current tobacco smoking was found to be the only significant predictor of lower S100 levels. Importantly, the S100 values were influenced by neither the resuscitation-related characteristics nor the cardiac function.

**Table 3:** Serum S100 cut-off value of 0.37 μg/L at 48 h relative to clinical characteristics of the study population.

	**S100 level**		**Odds ratio** (95% confidence interval)^*^	***p*-Value**
	**≥0.37 μg/L**	**<0.37 μg/L**		
	***n* = 7**	***n* = 41**		
**Clinical characteristics**				
Age (years)	65 ± 14	61 ± 12	1.0 (1.0–1.1)	0.41
Male	6 (86)	34 (83)	1.2 (0.1–12)	0.85
**Comorbidities**				
Diabetes mellitus	0 (0)	7 (17)	0.3 (0.0–6.0)	0.57
Arterial hypertension	6 (86)	25 (61)	3.8 (0.4–35)	0.21
Smoking	0 (0)	22 (54)	0.1 (0.0–1.1)	0.01
Dyslipidemia	3 (43)	13 (32)	1.6 (0.3–8.3)	0.67
Coronary artery disease	2 (29)	10 (24)	1.2 (0.2–7.4)	1.00
**Biomarkers**				
Lactate at baseline (mmol/L)	4.4 [2.8–15]	3.0 [2.5–5.6]	1.2 (1.0–1.6)	0.13
Hs troponin T at baseline (ng/L)	107 [78–332]	128 [49–244]	1.0 (1.0–1.0)	0.53
**Left ventricular ejection fraction (%)**	41 ± 14	35 ± 12	1.0 (1.0–1.1)	0.23
**Resuscitation-related characteristics**				
Witnessed OHCA	7 (100)	36 (88)	2.3 (0.1–45)	1.00
Basic life support performed	6 (86)	34 (83)	1.2 (0.1–12)	1.00
Basic life support time (min)	12 [8–13]	9 [6–10]	1.2 (0.9–1.7)	0.14
VT/VF upon first contact	4 (57)	34 (83)	0.3 (0.0–1.5)	0.15
Advanced life support time (min)	15 [15–45]	18 [10–21]	1.0 (1.0–1.1)	0.52
Time from OHCA to ROSC (min)	27 [22–54]	21 [16–30]	1.1 (1.0–1.2)	0.09
Normal pupillary reflex at baseline	6 (86)	39 (95)	0.3 (0.0–3.9)	0.38
**Electrocardiography**				
ST-segment elevation or LBBB	4 (57)	20 (49)	1.4 (0.3–7.1)	1.00
ST-segment depression	4 (57)	14 (34)	2.6 (0.5–13)	0.40
Right bundle branch block	2 (29)	4 (10)	3.7 (0.5–26)	0.21
No ischemic ECG changes	1 (14)	8 (20)	0.7 (0.1–6.5)	1.00
**Temperature management**				
Body temperature at baseline (°C)	35.4 ± 1.0	35.7 ± 0.7	0.5 (0.1–1.7)	0.33
Body temperature during TTM (°C)	34.3 ± 0.7	34.4 ± 0.6	0.7 (0.2–2.8)	0.58
Time from OHCA to target range (min)	97 [71–151]	101 [65–177]	1.0 (1.0–1.0)	0.98
Time from ROSC to target range (min)	53 [40–104]	75 [42–160]	1.0 (1.0–1.0)	0.55
Time within the temperature range (h)	20 [14–26]	22 [15–26]	1.0 (0.9–1.2)	0.92
**Cardiology intensive care unit care**				
Revascularization immediate (≤ 2 h)	1 (14)	17 (41)	0.2 (0.0–2.1)	0.23
Coronary angiography not performed	3 (43)	3 (7)	9.5 (1.4–64)	0.03
Need for vasopressors	6 (86)	19 (46)	6.9 (0.8–63)	0.10
Length of stay (days)	5 [3–6]	6 [5–8]	0.8 (0.5–1.1)	0.23
**Final diagnosis**				
Myocardial infarction	3 (50)	29 (71)	0.4 (0.1–2.3)	0.37
Cardiomyopathy	0 (0)	4 (10)	0.6 (0.0–11)	1.00
Primary arrhythmogenic cause	0 (0)	2 (5)	1.1 (0.0–24)	1.00
Extra-cardiac	1 (14)	4 (10)	1.5 (0.1–16)	0.56

The values are count (%), median [Q1–Q3], or mean ± standard deviation.

* *Odds ratios and confidence intervals were calculated for S100 levels ≥0.37 μg/L. For continuous variables, unit odds ratios for 1.0 increments with corresponding confidence intervals are given. Haldane-Anscombe correction was used in the case of a zero cell in the contingency tables, therefore confidence interval can contain 1.0 despite p < 0.05. ECG, electrocardiogram; hs, high sensitivity; LBBB, left bundle branch block; OHCA, out-of-hospital cardiac arrest; ROSC, return of spontaneous circulation; TTM, targeted temperature management; VT/VF, ventricular tachycardia/fibrillation*.

## Discussion

In a homogeneous population of OHCA victims undergoing TTM, this study found a significant association between 30 day neurological outcomes and S100 levels measured 48 h after the OHCA. However, the baseline values of S100 had no relationship to outcome. In fact, the 48 h S100 was the only independent predictor of neurological outcome. Using a cut-off value of ≥0.37 μg/L, S100 could predict an unfavorable outcome in 39% of patients with a 100% specificity. These findings suggest that the biomarker could be helpful for neurological prognostication, especially in patients who are still unconscious 48 h after their OHCA.

Dealing with comatose patients after resuscitation from an OHCA is notoriously difficult. Importantly, it is essential to minimize the risk of a falsely pessimistic prediction. When predicting poor outcomes, the false positive rate should be zero. Advantages of biomarkers over other prognostic modalities include quantitative results and likely independence from the effects of sedatives (10). In the central nervous system, the S100 family of proteins is present in glial and Schwann cells, where they stimulate cellular processes such as proliferation, differentiation, and regulation of intracellular Ca^2+^ homeostasis (38, 39). Extra-cerebrally, S100 is found in muscle, chondrocytes (40), and adipocytes (41). The latter sources may be confounding in OHCA patients who received chest compressions since they can lead to high baseline S100 levels. This increase was independent of the neurological outcomes in our patients.

The association between 48 h S100 serum levels and neurological outcomes in our cohort corresponds to previous observational trials, in which S100 measured at various time points after an OHCA were predictive of the neurological outcome (11–17, 19, 20, 24, 25, 27–30); nonetheless, only a few studies showed a significant association when the multivariate analysis was applied (23, 24, 26, 30). All the published evidence, to date, has significant shortcomings, including heterogeneous populations (11–16), or lack of routine TTM after OHCA, which is the current standard of care (16–26).

Previously, a biobank sub-study of the “TTM trial” analyzed serum S100 values of 687 patients after an OHCA (27, 35). After adjusting for relevant clinical factors, the authors found no independent predictive value of S100 measured at 24, 48, or 72 h. The sub-study compared S100 levels with a definitive 6 month CPC score, including all-cause mortality. However, this is a weak point of the observation, since by default, S100 levels cannot be used to predict all-cause mortality. To overcome this bias in analysis, our study considered the best CPC score, both during hospitalization and post-hospitalization, starting on day 3 and continuing through day 30. For instance, two of our patients, who had neurologically recovered during the first days (both were CPC 1), subsequently died from cardiac-related death; however, both were treated in the analysis as being CPC 1 (Figure 1). On the other hand, none of our patients who achieved a CPC score of 1 or 2 died during the 30 day study due to brain injury. We believe that our clinically more relevant and straightforward definition of the *neurological endpoint* was the main reason why our results differ from the “TTM trial” sub-study.

Our study has limitations. The required study population size was calculated based on previously published data. Although the study showed a significant association between high S100 levels and unfavorable neurological outcomes, the relatively modest sample size translates into wider CIs in the statistical analyses. The patient population was biased toward cardiac causes of OHCA since patients with obvious extra-cardiac causes of OHCA were predominantly transported to other intensive care units. On the other hand, this referral bias could be considered as an advantage since our study population was very homogeneous. Therefore, the study results are better applicable to CICU clinical practice. Finally, our results may be influenced by the automated method of electrochemiluminescence immunoassay using Elecsys S100® kits, which is more sensitive and has a faster turnaround time compared to kits of some other manufacturers (42).

This study showed that S100 values examined 48 h after OHCA can independently estimate 30 day neurological outcomes in a population of patients treated with TTM. Most importantly, this predictive value can be achieved without interference with TTM-associated sedation. Thus, the biomarker S100 may be particularly helpful in patients who remain comatose after completion of TTM. However, before making any critical clinical decisions, a neurological prognosis in these patients should be assessed by a multimodal approach, which should be based on repeated neurological assessments, and may include imaging, somatosensory evoked potentials, electroencephalography, and quantitative pupillometry (10, 43). In this regard, evaluation of S100 levels at 48 h would be a useful tool, though it is only one piece in the clinical mosaic.

## Data Availability Statement

The raw data supporting the conclusions of this article will be made available by the authors, without undue reservation.

## Ethics Statement

The studies involving human participants were reviewed and approved by Ethics Committee of the Institute for Clinical and Experimental Medicine and Thomayer Hospital, Prague. Written informed consent for participation was not required for this study in accordance with the national legislation and the institutional requirements.

## Author Contributions

MK: formal analysis, data curation, writing—original draft, and visualization. MS: data curation, writing—review and editing, and visualization. JKo: investigation. JKa: writing—review and editing. JKe: conceptualization, methodology, resources, supervision, and project administration. All authors contributed to the article and approved the submitted version.

## Conflict of Interest

JKa has received speaker's honoraria from Boehringer Ingelheim, Biosense Webster, Biotronik, Daiichi Sankyo, Medtronic, Merck Sharp & Dohme, Pfizer, and Abbott - St. Jude Medical; and has served as a consultant for Bayer, Boehringer Ingelheim, Biosense Webster, Daiichi Sankyo, Medtronic, Merit Medical, and Abbott - St. Jude Medical. The remaining authors declare that the research was conducted in the absence of any commercial or financial relationships that could be construed as a potential conflict of interest.
